# TF-Marker: a comprehensive manually curated database for transcription factors and related markers in specific cell and tissue types in human

**DOI:** 10.1093/nar/gkab1114

**Published:** 2021-11-19

**Authors:** Mingcong Xu, Xuefeng Bai, Bo Ai, Guorui Zhang, Chao Song, Jun Zhao, Yuezhu Wang, Ling Wei, Fengcui Qian, Yanyu Li, Xinyuan Zhou, Liwei Zhou, Yongsan Yang, Jiaxin Chen, Jiaqi Liu, Desi Shang, Xuan Wang, Yu Zhao, Xuemei Huang, Yan Zheng, Jian Zhang, Qiuyu Wang, Chunquan Li

**Affiliations:** School of Medical Informatics, Daqing Campus, Harbin Medical University. Daqing 163319, China; The First Affiliated Hospital, Institute of Cardiovascular Disease, Hengyang Medical School, University of South China, Hengyang, Hunan 421001, China; School of Medical Informatics, Daqing Campus, Harbin Medical University. Daqing 163319, China; State Key Laboratory of Genetic Engineering, Human Phenome Institute and School of Life Sciences, Fudan University, Shanghai 200438, China; School of Medical Informatics, Daqing Campus, Harbin Medical University. Daqing 163319, China; School of Medical Informatics, Daqing Campus, Harbin Medical University. Daqing 163319, China; School of Medical Informatics, Daqing Campus, Harbin Medical University. Daqing 163319, China; School of Medical Informatics, Daqing Campus, Harbin Medical University. Daqing 163319, China; School of Medical Informatics, Daqing Campus, Harbin Medical University. Daqing 163319, China; School of Medical Informatics, Daqing Campus, Harbin Medical University. Daqing 163319, China; School of Medical Informatics, Daqing Campus, Harbin Medical University. Daqing 163319, China; School of Medical Informatics, Daqing Campus, Harbin Medical University. Daqing 163319, China; School of Medical Informatics, Daqing Campus, Harbin Medical University. Daqing 163319, China; School of Medical Informatics, Daqing Campus, Harbin Medical University. Daqing 163319, China; School of Medical Informatics, Daqing Campus, Harbin Medical University. Daqing 163319, China; School of Medical Informatics, Daqing Campus, Harbin Medical University. Daqing 163319, China; The First Affiliated Hospital, Institute of Cardiovascular Disease, Hengyang Medical School, University of South China, Hengyang, Hunan 421001, China; School of Computer, University of South China, Hengyang, Hunan 421001, China; The First Affiliated Hospital, Cardiovascular Lab of Big Data and Imaging Artificial Intelligence, Hengyang Medical School, University of South China, Hengyang, Hunan 421001, China; Hunan Provincial Base for Scientific and Technological Innovation Cooperation, University of South China, Hengyang, Hunan 421001, China; The First Affiliated Hospital, Institute of Cardiovascular Disease, Hengyang Medical School, University of South China, Hengyang, Hunan 421001, China; School of Computer, University of South China, Hengyang, Hunan 421001, China; The First Affiliated Hospital, Cardiovascular Lab of Big Data and Imaging Artificial Intelligence, Hengyang Medical School, University of South China, Hengyang, Hunan 421001, China; Hunan Provincial Base for Scientific and Technological Innovation Cooperation, University of South China, Hengyang, Hunan 421001, China; School of Medical Informatics, Daqing Campus, Harbin Medical University. Daqing 163319, China; The First Affiliated Hospital, Institute of Cardiovascular Disease, Hengyang Medical School, University of South China, Hengyang, Hunan 421001, China; School of Computer, University of South China, Hengyang, Hunan 421001, China; The First Affiliated Hospital, Cardiovascular Lab of Big Data and Imaging Artificial Intelligence, Hengyang Medical School, University of South China, Hengyang, Hunan 421001, China; Hunan Provincial Base for Scientific and Technological Innovation Cooperation, University of South China, Hengyang, Hunan 421001, China; The First Affiliated Hospital, Institute of Cardiovascular Disease, Hengyang Medical School, University of South China, Hengyang, Hunan 421001, China; School of Computer, University of South China, Hengyang, Hunan 421001, China; The First Affiliated Hospital, Cardiovascular Lab of Big Data and Imaging Artificial Intelligence, Hengyang Medical School, University of South China, Hengyang, Hunan 421001, China; Hunan Provincial Base for Scientific and Technological Innovation Cooperation, University of South China, Hengyang, Hunan 421001, China; State Key Laboratory of Genetic Engineering, Human Phenome Institute and School of Life Sciences, Fudan University, Shanghai 200438, China; School of Medical Informatics, Daqing Campus, Harbin Medical University. Daqing 163319, China; The First Affiliated Hospital, Institute of Cardiovascular Disease, Hengyang Medical School, University of South China, Hengyang, Hunan 421001, China; School of Medical Informatics, Daqing Campus, Harbin Medical University. Daqing 163319, China; School of Computer, University of South China, Hengyang, Hunan 421001, China; The First Affiliated Hospital, Cardiovascular Lab of Big Data and Imaging Artificial Intelligence, Hengyang Medical School, University of South China, Hengyang, Hunan 421001, China; Hunan Provincial Base for Scientific and Technological Innovation Cooperation, University of South China, Hengyang, Hunan 421001, China; School of Medical Informatics, Daqing Campus, Harbin Medical University. Daqing 163319, China; The First Affiliated Hospital, Institute of Cardiovascular Disease, Hengyang Medical School, University of South China, Hengyang, Hunan 421001, China; School of Computer, University of South China, Hengyang, Hunan 421001, China; The First Affiliated Hospital, Cardiovascular Lab of Big Data and Imaging Artificial Intelligence, Hengyang Medical School, University of South China, Hengyang, Hunan 421001, China; Hunan Provincial Base for Scientific and Technological Innovation Cooperation, University of South China, Hengyang, Hunan 421001, China; General Surgery Department, Beijing Friendship Hospital, Capital Medical University, Beijing 100050, China; Guangxi Key Laboratory of Diabetic Systems Medicine, Guilin Medical University, Guilin, Guangxi 541199, China

## Abstract

Transcription factors (TFs) play key roles in biological processes and are usually used as cell markers. The emerging importance of TFs and related markers in identifying specific cell types in human diseases increases the need for a comprehensive collection of human TFs and related markers sets. Here, we developed the TF-Marker database (TF-Marker, http://bio.liclab.net/TF-Marker/), aiming to provide cell/tissue-specific TFs and related markers for human. By manually curating thousands of published literature, 5905 entries including information about TFs and related markers were classified into five types according to their functions: (i) TF: TFs which regulate expression of the markers; (ii) T Marker: markers which are regulated by the TF; (iii) I Marker: markers which influence the activity of TFs; (iv) TFMarker: TFs which play roles as markers and (v) TF Pmarker: TFs which play roles as potential markers. The 5905 entries of TF-Marker include 1316 TFs, 1092 T Markers, 473 I Markers, 1600 TFMarkers and 1424 TF Pmarkers, involving 383 cell types and 95 tissue types in human. TF-Marker further provides a user-friendly interface to browse, query and visualize the detailed information about TFs and related markers. We believe TF-Marker will become a valuable resource to understand the regulation patterns of different tissues and cells.

## INTRODUCTION

Marker genes are signatures in specific cell and tissues. And marker genes can be also used as biomarkers in certain diseases, while cell/tissue-specific marker genes can also help automatically annotate cell types in single-cell sequencing technology. Transcription factors (TFs) can recognize and bind to specific DNA sequences to guide expression of cell marker genes and maintain cell identity. Marker genes can enhance the ability to characterize cell types (1–4). Cell/tissue-specific TFs have multiple relationships with markers. For example, TFs can regulate the expression of cell markers (5–7). FOXA1 (HNF3A) is a TF involved in embryonic development which plays an important role in cancer. Studies have shown that FOXA1 could regulate the expression of cell marker PLOD2 by binding to promoters, thereby affecting the occurrence and development of lung cancer (8). TF POU5F1 controls the expression of a number of cell markers (e.g. YES1, FGF4, UTF1 and ZFP206) involved in embryonic development, which is critical for early embryogenesis and embryonic stem cell pluripotency (9). Furthermore, the expression of cell markers can influence the activity of TFs. Dang *et al.* evaluated the role of CD27 in inducing the expression of TFs (PRDM1 and XBP1) involved in plasma cell differentiation. They demonstrated that CD27 could activate PRDM1 and XBP1 by binding to CD70 on B cells (10). Moreover, a number of TFs play crucial roles as verified cell markers or potential markers in biological processes. TF GATA3 is a definitive cell marker of breast cancer. Visvader *et al.* identified GATA3, which promoted the differentiation of progenitor cells, as an important marker of tumor initiation (11). Becker *et al.* (12) found that TF LGR5 was a potential marker of intestinal stem cells in human. Overall, a variety of relationships between TFs and related markers have been confirmed by low-throughput biological experiments such as quantitative reverse transcription-polymerase chain reaction (qRT-PCR), western blot, knock down and luciferase reporter assays (13,14).

At present, some databases have been published for TFs or markers. For example, CellMarker (16) has been established to collect cell/tissue-specific cell markers for human and mouse. The data of CellMarker were collected from the PubMed database, handbooks and instructional websites from eight companies (Bio-Rad, Labome, BD Biosciences, R&D Systems, BioLegend, Abcam, Miltenyi Biotec and Thermo Fisher Scientific). MarkerDB (17) consolidates information on clinical and a selected set of pre-clinical molecular biomarkers for human disease into a single resource. However, these existing cell marker databases do not fully focus on cell/tissue-specific TFs and TF-related markers backed by experimental evidence. Other databases and algorithms such as TRANSFAC (18), JASPAR (19), TFCat (20), AnimalTFDB (21), TcoF-DB (22), KnockTF (23), WSMD (24) and TFBSImpute (25) have been developed for TFs and provide a resource for the expression, interactions and functions of TFs. These databases have also become valuable resources for TF research. For example, ReMap (26) is a database which provides the largest catalog of high-quality regulatory regions from an integrative analysis. ReMap helps researchers analyze the regulatory relationships between TFs and marker genes. Expression Atlas (27) is an added-value database that provides information about gene and protein expression in different species and contexts, such as tissue, developmental stage, disease or cell type. However, these databases do not fully explore the links betwteen TFs and cell markers. A large number of studies have shown that human TFs play crucial roles as cell markers in specific cells and tissues. More importantly, TFs and related markers have multiple relationships in specific cells and tissues. Therefore, it is highly desirable to construct a comprehensive resource of manually curated human TFs and related markers which provides comprehensive experimental evidence.

**Figure 1.**
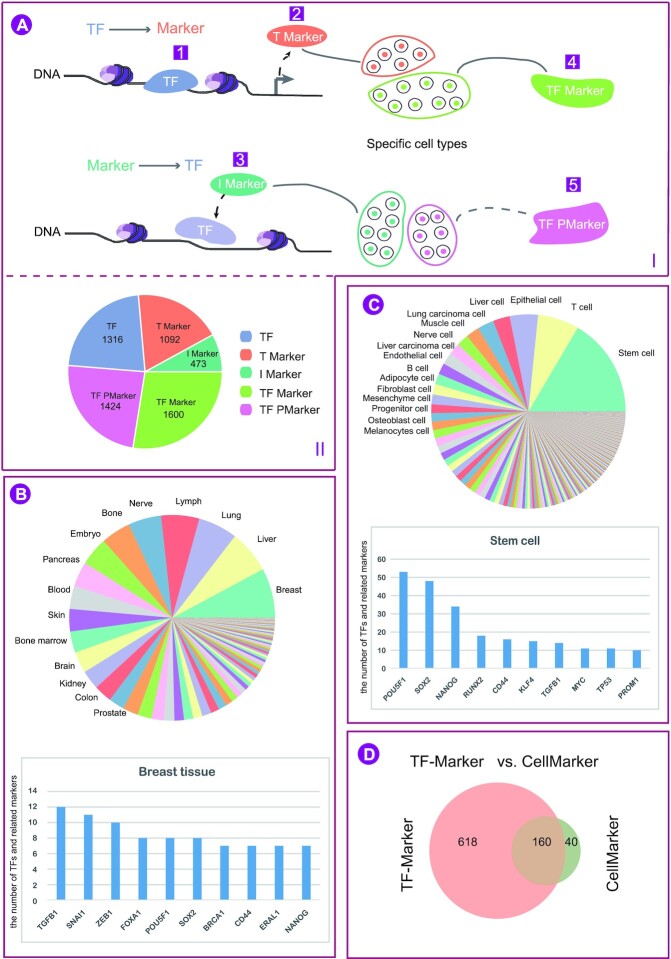
Statistics of TFs and related markers in TF-Marker. (**A**I) TFs and related markers were classified into five types according to their functions: one TF: TFs which regulate expression of the markers; two T Marker: markers which are regulated by the TF; three I Marker: markers which influence the activity of TFs; four TFMarker: TFs which play roles as markers and five TF Pmarker: TFs which play roles as potential markers. (**A** II) Number of TFs and related marker entries in TF-Marker. (**B**) The top 15 tissue types ranked by the number of entries in TF-Marker, and the top 10 TFs and related markers in breast tissue. (**C**) The top 15 cell types ranked by the number of entries in TF-Marker. The top 10 TFs and related markers in stem cells. (**D**) TF-Marker includes 80% of TFs in listed in CellMarker, which were collected from experiments and reviews.

Here, we developed the TF-Marker database (TF-Marker, http://bio.liclab.net/TF-Marker/) which is committed to a comprehensive manual curation of TFs and related markers with experimental evidence in specific cell and tissue types in human. Currently, through reviewing 2,091 published literature, we have manually classified TFs and related markers into five types according to their functions: (i) *TF*: TFs, which regulate the expression of markers. For example, FOXA1 (HNF3A) is a TF involved in embryonic development which plays an important role in cancer. Studies showed that FOXA1 could regulate the expression of cell marker PLOD2 by binding to its promoters, thereby affecting the occurrence and development of lung cancer (Figure 1 A 1); (ii) *T Marker*: markers, which are regulated by TFs. For example, TF POU5F1 controls the expression of marker genes (e.g. YES1, FGF4, UTF1 and ZFP206) involved in embryonic development. These genes are defined as Tmarker (Figure 1 A 2); (iii) *I Marker*: markers, which influence the activity of TFs. For example, Marker gene CD27 could activate PRDM1 and XBP1 by binding to TF CD70 on B cells. CD27 plays a role as I Marker (Figure 1A3); (iv) *TFMarker*: TFs, which play roles as markers. TFMarkers are usually cell/tissue-specific TFs and used as cell markers. For example, TF GATA3 is a definitive cell marker of breast cancer (Figure 1A4); and (v) *TF Pmarker*: TFs, which play roles as potential markers. For example, Becker *et al.* (12) found that TF LGR5 was a potential marker of intestinal stem cells in human (Figure 1A5). By curating thousands of published literature, 5905 entries including 1316 TFs, 1092 T Markers, 473 I Markers, 1600 TFMarkers and 1424 TF Pmarkers, were annotated in 383 cell types and 95 tissue types in human. Moreover, TF-Marker divided markers into disease markers and tissue/cell-specific markers. TF-Marker is an elaborate database, which provides TFs and related markers supported by experimental evidence. We believe that TF-Marker will provide strong support for research into cell/tissue-specific TFs and related markers.

## DATA COLLECTION AND DATABASE CONTENT

To ensure high quality data collection, we referred to the steps involved in the manual collection of other databases, such as CellMarker (14), ENdb (28) and EVLncRNAs (29). The literature with ‘transcription factor(s)’ was initially retrieved from the PubMed database. We found >70 000 literature with TFs and related markers mentioned in the titles or abstracts. Then, we carefully read these abstracts and retained >10 000 literature that included the relationships between TFs and markers. We applied the following workflow to obtain specific information about the TFs and related markers. First, we screened the literature based on two standards: (i) experimental evidence that could confirm the relationships between TFs and markers was mentioned in each article (e.g. qRT-PCR, western blot); and (ii) names of the cells or tissues were mentioned in the corresponding experiments. As a result, a total of 2091 literature were obtained according to our extraction requirements. Second, we carefully scrutinized the full text of the 2091 literature and obtained detailed information about the TFs and related markers. This detailed information included the PubMed ID of the literature, gene name, gene type (TF, T Marker, I Marker, TFMarker or TF Pmarker), detailed description of the TFs and related markers, cell name, cell type, tissue type, experimental technique (e.g. qRT-PCR, western blot, knock down, luciferase reporter assay) and experiment type (‘low-throughput’ or ‘high-throughput’). Third, the information about TFs and related markers was further expanded and standardized. We normalized the official names of TFs and related markers from the Gene (http://www.ncbi.nlm.nih.gov/gene) and Ensembl databases (http://ensemblgenomes.org/), and provided Entrez and Ensembl gene ID. Furthermore, names of tissues were normalized into the standard tissue list from UniProt (9) and cell names were normalized into an integrated reference list based on the Human Cell Atlas (2) and CELLPEDIA (30). TF family information was provided by TFClass (31). We also obtained gene expression atlases from GTEx (32), CCLE (https://sites.broadinstitute.org/ccle/), TCGA (https://cancergenome.nih.gov/) and ENCODE. Finally, we obtained 5905 TFs and related markers involved in 383 cell types and 95 tissue types in human. For each literature, two biological researchers carefully read the full text and examined the information in the literature twice.

### The relationships between TFs, super enhancers (SEs) and marker genes

TF-Marker collected experimentally confirmed TFs and their related markers. In order to better understand the relationship between TFs and marker genes, we constructed TF-SE-Marker gene transcriptional regulatory relationships using SEanalysis (34), which was developed by our group. SEanalysis is an SE upstream and downstream transcription regulation analysis tool. The TF-SE pair is predicted based on two methods: (i) TF ChIP-seq data obtained from databases such as ReMap (26) and Cistrome (35); and (ii) Motif scanning based on FIMO. The SE-Marker gene pair is predicted based on four strategies: closest active genes (36), overlapping genes, proximal genes and the closest genes (37). In order to further understand the regulation of TF-SE-Marker gene, we have checked the interaction in SEanalysis. We provide the TF-SE-Marker gene pair and other information on the detail page.

### The core TFs in core transcriptional regulatory circuit (CRC)

The CRC is comprised of a group of interconnected auto-regulating TFs forming loops (38–40). The core TFs in CRCs have been shown to be important for cell type-specific transcriptional regulation in normal cells and disease cells (41,42). The core TFs in CRCs are expected to be a reference for markers used to identify specific cell types (38,43). Therefore, we determined the core TFs in CRCs by integrating human H3K27ac ChIP-seq data from SEdb (44). Specifically, we first collected H3K27ac ChIP-seq data from NCBI GEO (15), ENCODE (33), Roadmap (33,45) and GGR (33). Next, Bowtie (v0.12.9) (46,47) and MACS were used for sequence alignment and peak calling of the ChIP-seq data, respectively (48). Finally, all CRC and core TFs were predicted by ROSE (37), CRCmapper (36) and the Coltron (39) program using default parameters and were added to TF-Marker. Users can browse the information of the core TFs in CRCs in TF-Marker.

## DATABASE STATISTICS

The current version of TF-Marker includes 1316 TFs, 1092 T Markers, 473 I Markers, 1600 TFMarkers and 1424 TF Pmarkers, involving 95 tissue types and 383 cell types in human (Figure 1AII). The top 15 tissue types ranked by the number of entries in TF-Marker included breast, liver, lung, lymph, nerve and other tissues. (Figure 1B). TGFB1, SNAI1 and ZEB1 were the top three genes collected in breast tissue (Figure 1B). Inflammatory breast cancer cells are usually characterized by the expression of these three genes (49–51). Furthermore, the statistical results for cell types showed that stem cells were the top cell type ranked by the number of all entries in TF-Marker. The top ten TFs and related markers such as POU5F1, SOX2, NANOG, RUNX2 and CD44 have been extensively researched in stem cells (Figure 1C). The expression of these markers controls the cell phenotype (52). TF-Marker contains 778 TFs with experimental evidence, and also includes 80% of the TFs in CellMarker, which were collected from experiments and reviews (Figure 1D).

## USER INTERFACE

### User-friendly interface for browsing TFs and markers

**Figure 2.**
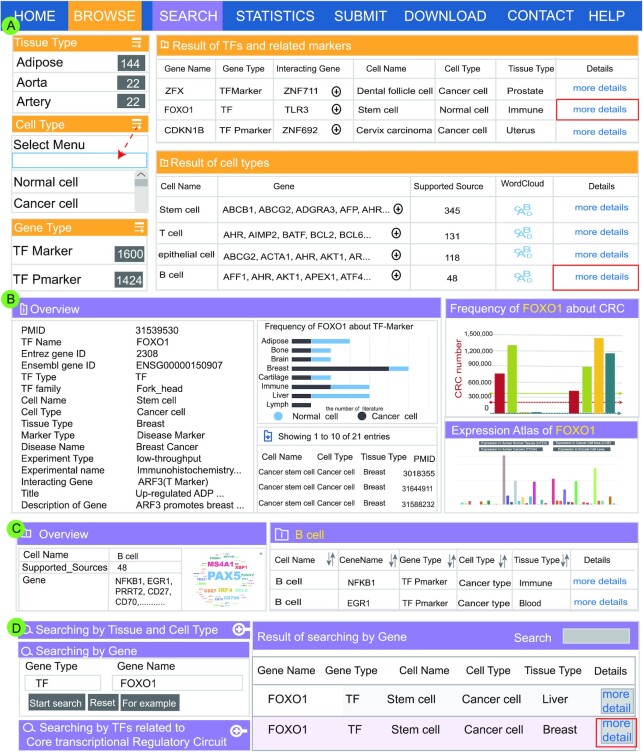
Main functions and usage of TF-Marker. (**A**) User-friendly interface for browsing TFs and markers. (**B**) The details of TFs and related markers. (**C**) Overview of details of TFs and related markers based on specific cell types. (**D**) Three paths for searching cell/tissue-specific TFs and related markers.

The ‘Browse’ page is organized as an interactive and alphanumerically sortable table that allows users to quickly browse through ‘Tissue Type’, ‘Gene Type’ and ‘Cell Type’. Users can browse TFs and related markers of interest via fuzzy search functions. TF-Marker presents two visual tables for users to view the information about TFs and related markers. One table is designed to list the information about multiple TFs and related markers via gene names. The other table is designed to display information about cell-specific TFs and related markers via cell types (Figure 2A). For browsing the details of TFs and related markers, users can click on ‘more details’. TF-Marker will return an overview of genes of interest (Figure 2B). Users can also obtain the list of TFs and related markers by selecting their corresponding cell types. Users can select tissue types and cell types to access TFs and related marker entries quickly. TF-Marker also adds a drop-down menu of ‘Show entries’ to change record numbers per page. Furthermore, users can click ‘more details’ to view details of TFs and related markers of interest (Figure 2C).

### Search interface for conveniently retrieving TFs and related markers

TF-Marker provides a convenient interface for retrieving genes on the ‘Search’ page. Users can search for information about TFs and related markers through three paths, including ‘Searching by Tissue and Cell Type’, ‘Searching by Gene’ and ‘Searching by TFs related to CRC’ (Figure 2D, left). Through ‘Searching by Gene’, the information about TFs and related markers can be obtained by inputting a single gene name (or gene alias) or gene lists. Brief information from the search results is then displayed in a table on the results page (Figure 2D, right). Clicking on ‘more details’, TF-Marker will display ‘PMID’, ‘Gene Name’, ‘Gene Type’, ‘Cell Name’, ‘Gene ID’, ‘Ensembl ID’, ‘TF Family’, ‘Tissue Type’, ‘Marker Type’, ‘Disease Name’, ‘Experimental name’, and a description of TFs and related markers in the literature. The detailed information can also be obtained by the visualization method. Additionally, TF-Marker display a graph to show entries of genes in different cell types of multiple tissues. The annotation information of TFs in CRCs is also presented on this page (Figure 2B). In addition, TF-Marker integrates the gene expression data of GTEx, CCLE, TCGA and ENCODE to display the expression levels of the gene. TF-Marker also provides users with more reference information such as TFs related to CRCs (Figure 2B).

### Download, submit and help interface

TF-Marker supports user downloads of all data and the submittal of new data via the ‘Download’ and ‘Submit’ page, respectively. The ‘Help’ page provides a detailed tutorial for users.

### Applications of TF-Marker

#### Case 1. Obtaining TFs and related markers in stem cells

**Figure 3.**
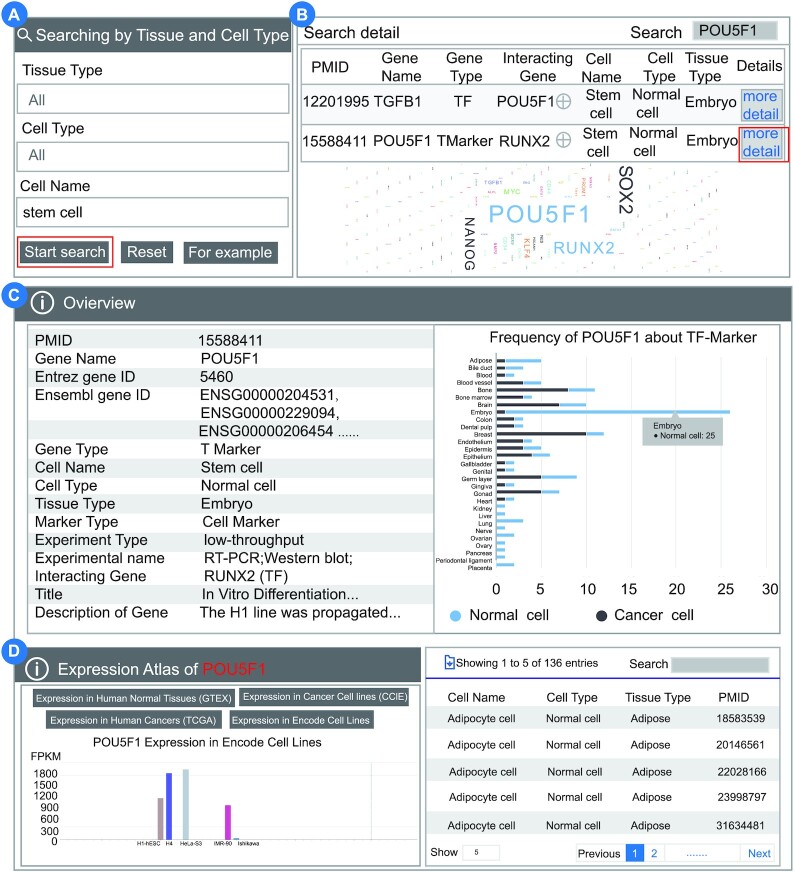
TFs and related markers in stem cells. (**A**) Searching for TFs and related markers in stem cells. (**B**) A summarized results table for stem cells. (**C**) The distribution graph is displayed based on the number of entries occupied by POU5F1 in the TF-Marker total results. The distribution shows 25 records of POU5F1 studies in embryo research. The list of the literature recorded in TF-Marker for POU5F1 is shown below. (**D**) The expression of POU5F1 in GTEx, CCLE, TCGA and ENCODE.

The TF-Marker database provides users with a function for obtaining TFs and related markers of interest in specific cell types of different tissues. Stem cells have major implications for research into developmental biology. As part of the process of cellular turnover and regeneration, stem cells are essential in the development of human tissues. Here, we applied TF-Marker to investigate the relevant TFs and related markers in stem cells. Users can select the cell name of ‘Stem cell’ in the ‘Searching by Tissue and Cell Type’ section (Figure 3A). TF-Marker will return a table and an intuitive summarized statistical graph of the prevalence of TFs and related markers, which shows all TFs and related markers of the stem cell. Users can download the list of TFs and related markers in stem cells by clicking the download button. Among these TFs and related markers, we found that POU5F1, SOX2 and RUNX2 were the top three genes investigated in stem cells. Users can extract the POU5F1-related contents by inputting POU5F1 in the search box, based on the total results. TF-Marker will display all the entries about POU5F1 based on the filter (Figure 3B). Users can click ‘more details’ to obtain the information about POU5F1. According to the search results, as a T Marker, POU5F1 is regulated by TF RUNX2 in embryo stem cells. The distribution is displayed based on the number of entries occupied by POU5F1 in the TF-Marker total results. It aims to show in which tissues and cells POU5F1 has been widely studied. The results indicate that POU5F1 is a widely studied marker in embryo tissue. The distribution shows 25 records of POU5F1 studies in embryo research (Figure 3C upper right panel). The list of the literature recorded in TF-Marker for POU5F1 is shown below (Figure 3C, bottom right panel). The expression of POU5F1 in GTEx, CCLE, TCGA and ENCODE is also displayed (Figure 3D).

#### Case 2. Searching for differential expressed TFs and related markers in breast tissue

**Figure 4.**
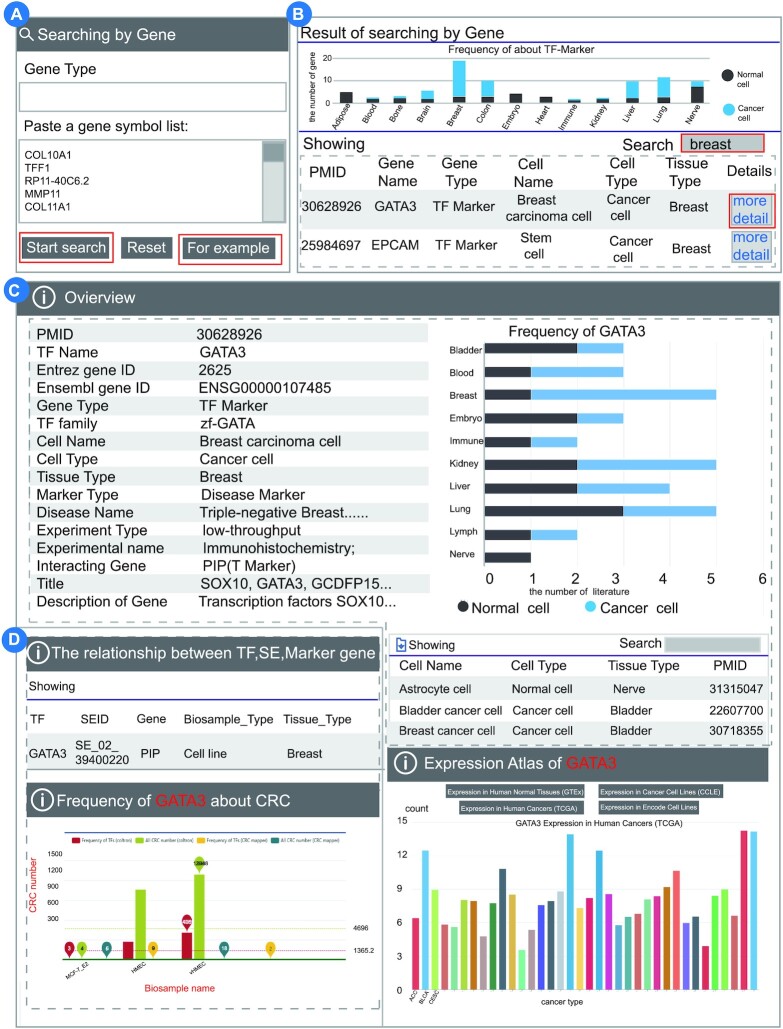
Differential expressed TFs and related markers in breast tissue. (**A**) Searching for differential expressed genes using the TF-Marker function ‘Searching by Gene’. (**B**) TF-Marker provides the distribution of the differential expressed genes in different tissues. (**C**) The detailed information of GATA3 is provided. (**D**) The information of GATA3 is displayed.

To further verify the function of TF-Marker, we downloaded gene expression data for breast cancer from TCGA and obtained 248 differential expressed genes with |log FC|>2 and *P* < 0.001. The 248 differential expressed genes were used as input for ‘Searching by Gene’ in TF-Marker (Figure 4A, Supplementary Table S1). Firstly, TF-Marker converted the gene alias to the official names when click the ‘Start search’ button. Then, the results of output will provide the distribution of the differential expressed genes in different tissues. As shown in the bar plot, we found that these genes were studied in multiple different tissues. Notably, the records of breast ranked first among all tissues. We extracted the breast-related contents by inputting ‘Breast’ in the search box. We could learn more about the function of GATA3 in breast cancer when clicked on ‘more detail’ (Figure 4B). As shown, GATA3 is a TFMarker which can regulate the T Marker PIP in breast cancer. GATA3 is regarded as a biomarker in Triple-negative breast cancer (Figure 4C, left). The distribution indicates that GATA3 is a marker which has been widely studied in breast tissue (Figure 4C, upper right panel). The list of the literature recorded in TF-Marker for GATA3 is shown below (Figure 4C, bottom right panel). We also found that GATA3 can regulate PIP gene by SE (Figure 4D, upper left panel). Moreover, GATA3 was further identified as a core TF (Figure 4D, bottom left panel) and has high expression in breast cancer (Figure 4D, right). Taken together, TF-Marker can help better understand regulatory relationship in biological research.

## SYSTEM DESIGN AND IMPLEMENTATION

We developed the current version of TF-Marker using MySQL 5.7.27. TF-Marker runs on a Linux-based Apache web server. We utilized PHP 5.6.40 for sever-side scripting, Bootstrap v3.37 and JQuerry v2.1.1 for interactive interface building, and Echats for visualization. For better display, we recommend using a comprehensive web server that supports HTML5 standard, for example, Firefox, Google Chrome and Safari. The research community can freely access information in the TF-Marker database without registering or logging in. The web link for TF-Marker is http://bio.liclab.net/TF-Marker/. PHP program is provided in Github website (https://github.com/LicLab-bio/PHP/tree/master/TF-Marker). The underlying data has been released in (https://doi.org/10.5281/zenodo.5574651).

## DISCUSSION

TFs play crucial roles in biological processes and are usually used as cell markers. The emerging importance of TFs and related markers in identifying specific cell types in human diseases increases the need for a comprehensive collection of human TFs and related markers sets (1). Therefore, we established a comprehensive database called TF-Marker which manually curates TFs and related markers in specific cell and tissue types in human. In the field of transcriptional regulation, ReMap (26) provides the largest catalog of high-quality regulatory regions resulting from a large-scale integrative analysis of TFs and regulators from DNA-binding experiments. MarkerDB and CellMarker have been constructed for marker research. Compared with these databases, TF-Marker focuses on cell/tissue-specific TFs and TF-related markers with experimental evidence (Table 1). MarkerDB consolidates information on clinical and a selected set of pre-clinical molecular biomarkers into a single resource, but it does not contain markers identified by histological, flow cytometry or other experiments. TF-Marker provides this information and further includes markers identified by single-cell RNA sequencing for different cell types. Compared with MarkerDB and CellMarker, TF-Marker finely divided TFs and related markers into five types according to the descriptions of research conclusions. In addition, we also provide information for core TFs in CRCs and their expression atlas in different cell lines/tissues.

**Table 1.** Comparison of information in TF-Marker with other databases

Attribution	TF-Marker	CellMarker	MarkerDB
TF number^a^	778	200	Unknown
Gene type	√	–	–
Interacting gene^b^	√	–	–
Experiment type	√	√	–
Experiment name	√	–	–
Biomarkers^c^	√	–	√
Description of the literature	√	–	–
Cell name	√	√	–
Cell type	√	√	–
Tissue Type	√	√	–
Expression atlas^d^	√	–	–
TF-SE-Marker gene^e^	√	–	–
The core TFs in CRCs^f^	√	–	–

^a^TF Number was the experimentally verified TFs for human.

^b^Interacting Gene was the genes that have some relationship in the biology process with the TFs or markers.

^c^In biological research, marker genes can be used as biomarkers in certain diseases.

^d^TF-Marker provides users with more TF reference information like expression atlas from GTEx, CCLE, TCGA (https://cancergenome.nih.gov/) and ENCODE.

^e^TF-SE-Marker gene regulation was constructed by SEanalysis.

^f^The core TFs in CRCs were determined by integrating human H3K27ac ChIP-seq data from SEdb.

The main advantages of the database are illustrated below: (i) TF-Marker provides comprehensive TF and related marker reference sets with classifications of TFs and related markers. We divided the TFs and related markers into five types according to their functions (TF, T Marker, I Marker, TFMarker and TF Pmarker). (ii) TF-marker is dedicated to collecting cell/tissue-specific TFs and related markers backed by experimental evidence. (iii) TF-Marker supports CRC TFs which have been proven highly valuable for understanding cell type-specific transcriptional regulation in normal and disease cells; (iv) TF-Marker supports the use of specific experimental names and descriptions of TFs and related markers; (v) TF-Marker provides relevant descriptions of TFs and markers in each published article; (vi) Users can obtain their TFs of interest and related markers by different searching interfaces; (vii) TF-Marker provides users with more TF reference information from expression atlases such as GTEx, CCLE, TCGA (https://cancergenome.nih.gov/) and ENCODE.

The current version of TF-Marker curates 5905 TFs and related markers. Through building a comprehensive database for TFs and related markers in various cells/tissues, TF-Marker contributes by advancing objective research into cell markers, while classification of markers through reading of the literature is subjective. We focus on the experimentally verified TFs and related markers, and the genes related to human diseases usually experimented on model organisms. We also collected them and normalized official names of TFs and related markers from Gene (http://www.ncbi.nlm.nih.gov/gene) and Ensembl database (http://ensemblgenomes.org/). With the development of single-cell sequencing technology and accumulation of experimental data, extensive literature regarding TFs and related markers will become available. We will manually curate these data in order to update this database in a timely manner. Furthermore, TF-Marker will be supplemented with additional functional information for TFs and related markers. TF-Marker will strive to expand upon the number of species and collections, including additional experimental methods to extend our data sources, constructing networks of TFs and disease-related markers, and providing users with powerful analysis tools in future versions. We believe this first version and future updates of TF-Marker will provide biologists with accessible information for research into different diseases.

## Supplementary Material

gkab1114_Supplemental_File
